# Color vision restrictions for medical school admission: a discussion on regulations in ASEAN countries compared to countries across the world

**DOI:** 10.1186/s40942-023-00441-4

**Published:** 2023-01-30

**Authors:** Ting Fang Tan, Andrzej Grzybowski, Paisan Ruamviboonsuk, Anna C. S. Tan

**Affiliations:** 1 Singapore National Eye Centre, Singapore General Hospital, Singapore, Singapore; 2 Department of Ophthalmology, Institute for Research in Ophthalmology, Foundation for Ophthalmology, University of Warmia and Mazury, Poznan, Poland; 3 Department of Ophthalmology, College of Medicine, Rangsit University, Rajavithi Hospital, Bangkok, Thailand; 4 Singapore National Eye Centre, Singapore Eye Research Institute, Duke-NUS, Singapore, Singapore

**Keywords:** Color vision, Medical school, Regulations

## Abstract

Color vision deficiency impairs one’s ability to perceive and discriminate colors. Color-deficient individuals may face discrimination in various occupations, particularly in medical school admissions. This discussion seeks to compare the existing color vision requirements for entry to medical school in Southeast Asian countries as compared to countries across the world. Following this, we explore the published evidence in this field, to provide recommendations for future guidelines that will maximize the occupational opportunities for color-deficient individuals.

## Introduction

Color vision deficiency can be congenital or acquired. Males (about 8%) are more commonly affected for the former as compared to their female counterparts (0.4%) [26]. Acquired causes can be a result of different etiologies including inflammatory diseases, medication toxicity, glaucoma, age-related macular degeneration, retinitis pigmentosa, or neurological causes like tumor or stroke [19].

Color vision deficiency is a spectrum of disease, hence the severity and impact on function vary between individuals. Normal individuals or normal trichromats have functioning cone photopigments in all three classes. For anomalous trichromats, one of the three cone photopigments has an abnormal absorption spectrum— tritanomaly, deuteranomaly and protanomaly for blue, green, or red photopigment anomaly respectively. For dichromats, one of the three cone photopigments is absent—tritanopia, deuteranopia and protanopia for absent blue, green or red photopigment respectively. Monochromats possess one or no functional cone photopigment [3].

Some professions rely on discrimination of colors more than others [11]. It has been demonstrated that color-deficient individuals face more difficulties in color-dependent tasks such as identifying traffic signal controls [48] and color-coded equipment [44]. In particular, it is not surprising for the medical profession to demand astute color discrimination from its physicians [13, 47]. Furthermore, as medical management of every patient is guided by clinical judgment, this has significant safety implications to the community at large.

To our knowledge, there has been limited literature published in the areas of color vision deficiency and medical school admissions originating from Southeast Asia. Therefore, in this study, we aim to outline the existing requirements for admission to medical schools among the Association of Southeast Asian Nations (ASEAN) ten member countries, namely Brunei, Cambodia, Indonesia, Laos, Malaysia, Myanmar, Philippines, Singapore, Thailand and Vietnam; as compared to countries across the world. We then explore the existing evidence on the impact of color vision deficiency on medical practice, to explore further recommendations on current requirements.

## Existing regulations for medical school admissions

Color vision deficiency is not a criterion for rejection to study or practice medicine in most countries such as the United States and the United Kingdom [13]. Historically, Japan had the most restrictive policy, with 55.8% of medical universities excluding individuals with deficient color vision in 1986. This dropped to 2.3% with efforts by the Japanese Ophthalmologists Association in 1992, subsequently dropped after campaigning efforts by ophthalmologist/activist Yasuyo Takayanagi [16, 44]. Takyanagi demonstrated that individuals who did not pass mandatory Ishihara tests were still able to execute color-coded tasks, in a study where participants were able to connect color cords without error [45]. Screening of color vision deficiency is only practiced at one university in the United Kingdom, where it is a part of the Queen’s University of Belfast, Northern Ireland routine medical examinations, but normal color vision is not a criterion for entry [14, 22]. Similarly, the Medical Council of India followed the recommendations by the Supreme Court to drop restrictions on applicants with color vision deficiency—at the stage of admission, completion of study, and registration as a medical practitioner—ending its decades-old practice [46].

Among the ten ASEAN countries discussed (Table 1), most countries (seven out of ten) did not have color vision requirements for medical school admissions. Only Indonesia and Malaysia excluded medical school applicants with any color vision impairment. In particular, color vision deficiency is screened in Myanmar and Thailand, not as a criterion for medical school entry, but instead to make the individual aware of their color vision deficiency. In countries where color vision was tested, the Ishihara test plates were used.

**Table 1:** Color vision requirements for medical school admissions in ASEAN countries [42]

Country	Color vision test	Authorized persons licensed to conduct testing	Exclusion criteria for medical school admissions
Brunei	N/A	N/A	N/A
Cambodia	N/A	N/A	N/A
Indonesia	Ishihara	Physician or Ophthalmologist	Any color vision deficiency
Malaysia	N/A	N/A	N/A
Myanmar	Ishihara	Ophthalmologist	N/A (screened for awareness)
Laos	N/A	N/A	N/A
Philippines	N/A	N/A	N/A
Singapore	N/A	N/A	N/A
Thailand	Optional	Admission board or medical schools	N/A (screened for awareness)
Vietnam	N/A	N/A	N/A

## Effect of color vision deficiency on medical practice

Difficulties in medical practice that have been identified amongst color-deficient doctors [37] mainly involved the diagnostic process. These challenges included interpretation of physical signs in clinical examination [e.g. skin [1]—cyanosis (bluish color to the skin from low oxygen), jaundice (yellowish color to the skin from the build up of toxins), erythema (redness of the skin); bodily fluids [6, 32]—urine, feces, vomitus], tests/instruments [8, 23, 30, 35] (e.g. test strips, charts, slides, otoscope/ophthalmoscope). In particular, these difficulties may be accentuated in situations when observed signs are pivotal (i.e. next course of action depends solely on the interpretation of color); working conditions such as poor lighting; or general practitioners who function alone where second opinions are not readily available [37].

Furthermore, misinterpretation in some instances may potentially be dangerous—one study reported that feeding down incorrectly placed nasogastric tubes resulted in eleven deaths in a 2 years [27]. Checking for correct placement of nasogastric tubes involves interpreting pH indicator colored test strips of aspirates, where color-deficient doctors may potentially make errors. Otherwise, there is no clear evidence to show the association with increased medical errors.

However, clinical medicine encompasses interpretation of all factors in the diagnostic process, that need not rely solely on color discrimination. In most clinical situations, with increasing experience, various other factors including history taking and targeted physical examination are involved in reaching a diagnosis and formulating a management plan [14]. Hence, with proper training most doctors with color vision deficiency are still able to function effectively.

Interestingly, a sizeable proportion of color-deficient individuals are unaware of their deficiency [2, 35, 37, 39]. As a result, this group of doctors may only reactively learn from avoidable mistakes, whereas a preferable strategy in an individual who is aware of their color deficiency, is to pre-empt specific difficulties and take active steps to minimize errors. On the other hand, even those who are aware of their color deficiency may often be unaware of its extent [37, 38]; and some may be more anxious about the risk of error or lack confidence in practice which may instead compromise patient care [14, 25].

Hence, color vision testing at the point of admission to medical school may allow potential doctors with color vision deficiency to make informed decisions on whether to pursue a future in the medical profession. A study by Chan et al. looked at the chronological impact on development and difficulties faced by individuals with color vision deficiency from playschool age to school age and adulthood [10]. 77% of color-deficient individuals only knew about their condition after formal color vision testing in their later years, where they recounted retrospectively the difficulties faced in their growing years [43]. This suggests a role for early screening for color vision deficiency to increase awareness [18].

## Moving forward

Color-deficient individuals should not be denied medical school admission or a practicing medical license as adaptive compensatory strategies can be employed to overcome the difficulties faced. Studies have emphasized the role of pre-entry testing of color vision, not as a criterion for entry, but to raise awareness amongst applicants on their deficiency and acknowledge the potential challenges [7, 12, 13]. These studies have also highlighted the role of institutions to provide appropriate counselling on compensatory mechanisms and facilitate informed career choices in particular to medicine [35, 36, 38, 40, 43]. For instance, doctors with abnormal color vision can minimize errors by their choice of specialty [36], utilizing other sources of information that are not color-dependent, and optimizing observation conditions like ambient lighting brightness [38]. For example, Rubin et al. demonstrated the use of grayscale copies to remove confusing stain colors and accentuate the contrast in texture of tissues to ease interpretation of histology slides [34]. More recently, ongoing efforts include digitalizing pathology slides and optimizing them via personalizing color maps for individual pathologists’ visual systems [29]. With technological advancements in the medical field, the use of optical aids, advanced imaging techniques, artificial intelligence with deep learning algorithms in clinical practice to strengthen diagnostic assessment may further reduce the reliance on color vision in clinical examination [9, 15, 17, 24].

However, there is limited published evidence on specific advice for color-deficient individuals to adopt in the field of clinical practice. Raynor et al. surveyed on 33 UK medical schools and 154 acute trusts found that 16.7% screened for color vision deficiency and 50% made adaptations for these students. However, the support given was focused on examinations (e.g. extra time given to candidates during examinations) but does not improve obstacles faced in real-life clinical practice [31].

Evaluation of current practice also calls for better standardization of color vision tests. Amongst the wide range of color vision tests, there lacks existing evidence to support which test is the most accurate and practical. Despite the anomaloscope’s high sensitivity and specificity [4], often regarded as the gold standard [20, 28, 41], it is expensive and technically challenging to use hence limiting its feasibility for routine use. On the other hand, commonly used tests like the Ishihara pseudoisochromatic test have been reported to have poor correlation with the type and severity of color vision deficiency [3, 33]. This once again limits its use in reflecting the impact of color vision deficiency on the individual’s function.

There have been new systems proposed to quantify the severity of color vision deficiency and identify what can be considered safe or functional based on the requirements of specific color vision tasks in visually demanding occupations. For example, color vision categories proposed by Barbur et al. range from ‘Supernormal’ trichromatic color vision (CV0), ‘Normal’ trichromatic color vision (CV1), ‘Functionally normal’ trichromatic color vision (CV2), ‘Safe’ trichromatic color vision (CV3), ‘Poor’ red-green color vision (CV4) and ‘Severe red-green color deficiency (CV5); and ‘Supernormal’, ‘Normal’ and ‘Acquired color deficiency’ for yellow-blue color vision deficiency. 22% of deutans and 1% of protans were classified as ‘Safe’ or ‘Functionally normal’ under this system [5]. Similarly, the Color Assessment and Diagnosis (CAD) test was developed to establish the minimum requirements to carry out the most safety–critical task. The CAD test has been adopted in other fields like the United Kingdom Civil Aviation Authority for professional aviation pilots [5] and the Transport for London for underground train captains [21], where about one-third of applicants, who would otherwise be rejected under conventional tests, were now considered safe. These efforts highlight the need to further validate the use of these tests to redefine color vision requirements in the medical field and its sub-specialties, to potentially minimize unnecessary discrimination against color-deficient individuals who can fulfill these tasks safely.

## Conclusion

Overall, the current evidence does not support excluding color-deficient individuals from practicing medicine. However, while many studies have emphasized the need for screening to increase awareness, there currently lacks evidence-based, deficiency-specific advice for color-deficient individuals who may potentially experience difficulties. Moreover, it may be of value to further study the different demands of color vision in specific sub-specialities in medical or surgical practice to provide color-deficient individuals with better early career advice. Hence, this suggests, that more research is required in these areas to derive updated standardized guidelines and tangible evidence-based advice for medical schools admissions.

## Data Availability

No data were generated or analyzed in the presented research.
